# LUMP Is a Putative Double-Stranded RNA Binding Protein Required for Male Fertility in *Drosophila melanogaster*

**DOI:** 10.1371/journal.pone.0024151

**Published:** 2011-08-30

**Authors:** Charcacia Sanders, Dean P. Smith

**Affiliations:** Departments of Pharmacology and Neuroscience, University of Texas Southwestern Medical Center, Dallas, Texas, United States of America; University of Otago, New Zealand

## Abstract

In animals, male fertility requires the successful development of motile sperm. During *Drosophila melanogaster* spermatogenesis, 64 interconnected spermatids descended from a single germline stem cell are resolved into motile sperm in a process termed individualization. Here we identify a putative double-stranded RNA binding protein LUMP that is required for male fertility. *lump^1^* mutants are male-sterile and lack motile sperm due to defects in sperm individualization. We show that one dsRNA binding domains (dsRBD) is essential for LUMP function in male fertility. These findings reveal LUMP is a novel factor required for late stages of male germline differentiation.

## Introduction

Spermatogenesis is the process where male diploid spermatogonia develop into mature, haploid spermatozoa capable of fertilizing an oocyte [1]. In all animals, this process involves a series of tightly regulated stages that include mitotic proliferation, meiotic division, and extensive cellular remodeling. The first step in spermatogenesis is the division of a self-renewing germline stem cell to produce a spermatogonial cell. This cell subsequently undergoes a series of mitotic divisions to produce spermatocytes that enter meiosis [2]. Following meiosis the haploid spermatocytes undergo a series of morphological changes producing mature spermatozoa [3], [4].

Spermatogenesis has been extensively studied in Drosophila and mutations that affect each of the major steps in spermatogenesis have been described [reviewed in [5]. Germline stem cells located at the apical tip of the testes divide asymmetrically to produce a new germline stem cell and a founder gonialblast [2]. The gonialblast, enclosed in a two-cell syncytium cyst, undergoes four mitotic divisions to produce 16 primary spermatocytes that remain interconnected through cytoplasmic bridges as a result of incomplete cytokinesis [6]. The 16 primary spermatocytes undergo two meiotic divisions to give rise to 64 spermatids that elongate and separate into individualized spermatozoa through a poorly understood process called individualization [4]. During individualization protamines are incorporated into the chromatin resulting in nuclear condensation [7]. Additionally, in a process called individualization, the membrane of the syncytium is remodeled to enclose each sperm.

RNA interference (RNAi) has been implicated in normal male fertility. Failure to silence non-coding RNAs through PIWI causes a loss of germ line stem cells [8], [9]. Similarly, mutations in the Argonaute family members *aubergine* and *spindle-E* de-repress *Stellate* which results in intracellular crystals in male germ cells causing sterility [10]–[11]. *Stellate* silencing also requires Loquacious, a dsRNA binding protein that associates with Dicer-1 to process pre-miRNAs [12]. Ago-3, an Argonaute protein enriched in the germline, has been implicated in both germline stem cell maintenance and *Stellate* silencing [13]. Thus, several double-stranded binding proteins are required for normal male fertility.

Here, we describe the identification of an orphan Drosophila gene, *lump*, predicted to encode a dsRNA binding protein similar in domain structure to R2D2, a protein required for RNAi [14], [15]. We show that mutants in *lump* are not defective for RNAi, miRNA processing, or Stellate suppression, but are defective for late stage sperm development and male fertility. This gene product was independently identified as a male fertility factor by Gerbasi et al. [16].

## Results

### LUMP has two dsRNA binding domains but is not required for RNA interference or miRNA processing in vivo

**Figure 1:**
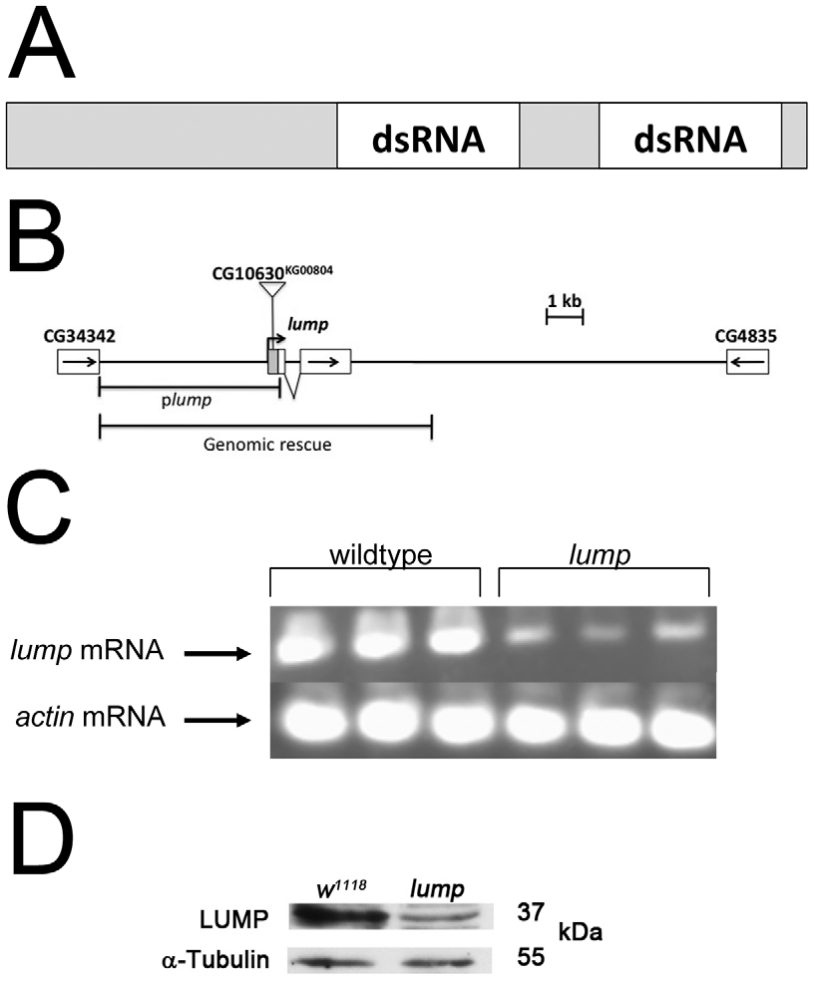
LUMP encodes a dsRNA binding domain protein. **A.** The LUMP protein has two putative dsRNA binding domains (dsRBD). **B**. Genomic structure of the *lump* locus. The triangle represents location of the P element insertion in *CG10630^KG00804^* in the 5′ UTR of *lump*. The arrows show the direction of transcription of *lump* and flanking genes. The genomic DNA used to drive expression in transgenic animals is depicted (genomic rescue) **C, D.** The P-element insertion causes a reduction of *lump* mRNA and protein levels in *lump^1^* mutants.

R2D2 is a 35 kD protein with two dsRNA binding domains and functions to facilitate loading of siRNA fragments from Dicer-2 to Ago-2 [14], [15], [17]. Mutants defective for R2D2 are defective for RNAi [15]. We searched the Drosophila genome for additional genes encoding potential dsRNA binding proteins. The predicted structure of LUMP (CG10630) is similar to R2D2, encoding two predicted dsRNA binding domains (Figure 1A) [18], [19]. To investigate the function of LUMP we set out to identify mutants defective for this protein. The P-element insertion strain CG10630^KG00804^, hereafter referred to as *lump^1^*, harbors a P-transposable element inserted in the 5′ UTR of the *lump* gene, 70 base pairs upstream of the coding region (Figure 1B). RT-PCR and western blot experiments confirm this allele is defective for expression of the *lump* gene. Semi-quantitative RT-PCR reveals a dramatic reduction in *lump* mRNA in *lump^1^* mutants (Figure 1C). Similarly, western blots using anti-peptide antiserum to LUMP indicate *lump^1^* is a strong hypomorph (Figure 1D). An expression survey reveals *lump* is normally expressed in many tissues at all developmental stages (Figure S1, Methods S1).

**Figure 2:**
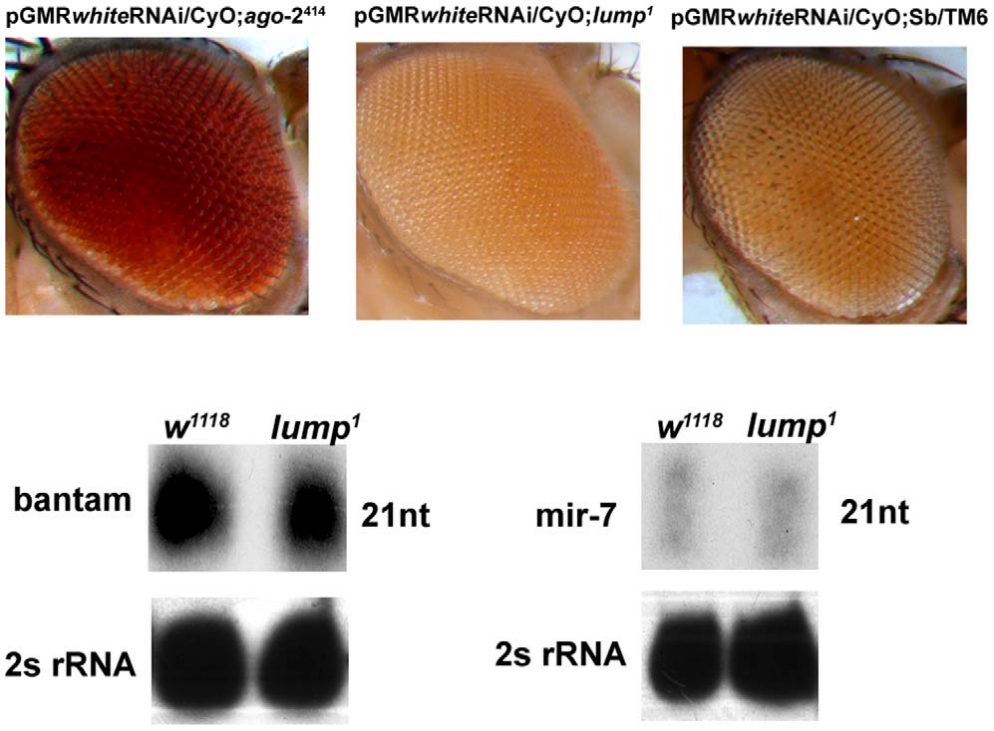
LUMP is not required for RNA interference or miRNA biogenesis. **Top Panels.** *Ago2^414^* mutants defective for RNAi and carrying one copy of pGMR-GAL4, UAS-*white*RNAi fail to suppress the *white* locus (left panel). RNAi is not blocked in *lump^1^* mutants carrying one copy of pGMR-GAL4, UAS-*white*RNAi (center panel). pGMR-GAL4, UAS-*white*RNAi in a wild type background (right panel). **Lower Panels.** Northern blots for *bantam* and *mir-7* miRNAs reveal no differences in quantity of mature miRNA in *lump^1^* mutant flies. 2S rRNA is a 30 nucleotide ribosomal RNA used as a loading control.

To determine if LUMP has a role in RNA interference, we examined the competence of *lump^1^* mutants to suppress RNAi *in vivo*. *white*RNAi is a genomic-cDNA fusion transgene that forms a double-stranded RNA in vivo [20]. When expressed with the eye-specific promoter, pGMR, *white* dsRNA suppresses expression of the *white* locus through RNA interference resulting in defective eye pigmentation [20]. One copy of *white*RNAi expressed under control of the pGMR reduces *white* expression approximately 90% in wild type flies, resulting in orange eyes [20]. This reporter system is extremely sensitive to changes in RNAi function. When one copy of pGMR-*white*RNAi is introduced into an RNAi-deficient *Ago-2* mutant background, RNAi is blocked and the eye color reverts to red (Figure 2). *lump^1^* mutants carrying pGMR-GAL4, UAS-*white*RNAi has wild type suppression of *white*. These results suggest that *lump^1^* mutants are not defective for RNA interference in vivo.

LUMP may function in an alternate RNAi pathway, such as the microRNA or PIWI silencing pathways, or mediate tissue-specific RNAi in other parts of the fly. If LUMP is important for miRNA processing or function, we expect to observe reduced levels of miRNA in *lump^1^* mutants. miRNAs are a large family of small, non-coding, endogenously produced RNAs and are key developmental regulators [21]–[22]. Mature miRNAs function to target the nuclease Ago-1 to specific mRNAs for translational repression [23]. Loquacious, a protein with three dsRNA binding domains, partners with Dicer-1 to facilitate processing of miRNAs. Mutants defective for *loquacious* are defective for miRNA processing but not RNAi [12].

We performed Northern blot analysis to examine the levels of *bantam* and *mir-7*, two microRNAs, in wild type and *lump^1^* mutants. We observe no difference in the mature miRNA levels in *lump^1^* mutants compared to controls (Figure 2, lower panels). This suggests that *lump^1^* mutants are not defective for biogenesis of these miRNAs.

### *lump^1^* mutants are sterile

The homozygous mutant *lump^1^* stock was unable to produce viable progeny, indicating a fertility defect. To confirm that the fertility defects result exclusively from the *lump-*associated P element, we mobilized transposon from the *lump* gene and recovered precise excisions. These revertants are fertile, clearly demonstrating the P element is indeed responsible for the fertility defect, and ruling out the possibility of a second site mutation producing sterility.

**Figure 3:**
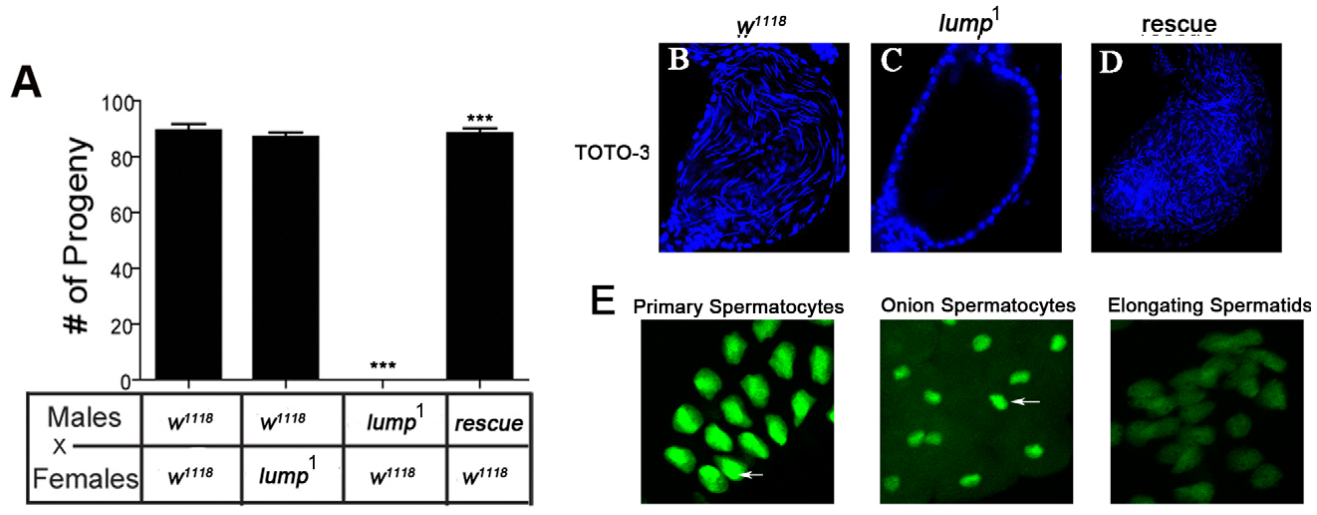
*lump^1^* mutants are male sterile. **A**. Number of progeny produced by males crossed to females of the depicted genotypes. *w^1118^* control males crossed to *lump^1^* mutant females produce similar numbers of progeny as wild type controls. *lump^1^* mutant males crossed to *w^1118^* control females are sterile. *lump^1^* males carrying a transgenic wild type copy of the *lump* gene (rescue) have fertility restored to wild type levels. All data are presented as ± SEM. For each data set statistical significance was tested using ANOVA for independent observations. *** represents significant differences at P < 0.001. **B**–**D**. TOTO-3 DNA staining of spermatid nuclei. **B**. TOTO-3 staining of sperm nuclei reveals *w^1118^* male seminal vesicles contain mature sperm. **C**. Seminal vesicles from *lump^1^* mutant males are devoid of intact spermatid nuclei. **D**. Seminal vesicles from *lump^1^* mutant males carrying a wild type transgenic copy of the *lump* gene contain mature sperm. **E**. GFP-LUMP expression in the testes. LUMP-GFP is expressed in spermatocyte nuclei of primary spermatocytes, in the nuclei and cytoplasm of onion stage spermatocytes and begins to degrade in elongating spermatids.

We next undertook experiments to establish whether the fertility of *lump^1^* males, females or both is compromised by the reduction in LUMP expression. Homozygous *lump^1^* mutant females produced a similar number of offspring as wild type controls when crossed to wild-type males (Figure 3A). However, no progeny were produced when homozygous *lump^1^* males (Figure 3A) or transheterozygous *lump^1^*/Df (3L)Exel6105 males [24], carrying a deletion that removes the *lump* locus (not shown), were crossed to control virgin females. Importantly, homozygous *lump^1^* males carrying a wild type transgenic copy of the *lump* gene have normal fertility (Figure 3A, rescue). Therefore, male fertility is specifically defective in *lump^1^* mutants, and this defect is exclusively due to loss of *lump* function.

### *lump^1^* males do not produce mature sperm

To investigate the male sterility associated with the loss of *lump* function, we analyzed male germ cells in these mutants. We asked whether *lump^1^* mutant males produce motile sperm. Seminal vesicles were dissected from both *lump^1^* and wild type males and stained with TOTO-3 DNA stain to identify sperm nuclei. Figure 3C shows that no mature sperm are present in seminal vesicles from *lump^1^* mutant males but are readily visible in seminal vesicles from wild type males (Figure 3A) or transgenic rescue flies (Figure 3D). Therefore *lump^1^* males fail to produce mature sperm.

### LUMP is expressed in spermatocytes

We analyzed the expression of *lump* to gain additional insight into its role in male fertility. The LUMP anti-peptide antiserum we used for Western blots failed to identify LUMP in tissues, therefore we generated p*lump*-Gal4 transgenic flies and LUMP-GFP fusion genes (see Materials and Methods). We crossed p*lump*-Gal4 flies to a reporter strain carrying UASp-LacZ. LacZ was expressed in the spermatocytes and elongating spermatids in the testes of these flies (not shown), indicating the *lump* gene is expressed in male germ cells.

We next examined the cellular and subcellular localization of LUMP in the testes. We produced transgenic flies that express a GFP-LUMP fusion protein expressed under direct control of the *lump* promoter (Figure 1B, Materials and Methods). The GFP-LUMP fusion reporter is functional, as it fully rescues the male fertility defects in *lump^1^* homozygous mutant males (see below). Figure 3E shows the GFP-LUMP reporter is localized to the nuclei of spermatocytes and is found in both the nuclei and the cytoplasm of onion stage spermatids. At later stages, the GFP-LUMP signal fades in elongated spermatid bundles and is absent from mature sperm. These data indicate that LUMP protein is transiently expressed in developing male germ cells, and shifts from a nuclear to cytoplasmic localization before being completely degraded prior to formation of individual mature spermatozoa.

### *lump^1^* mutant spermatids fail to individualize

**Figure 4:**
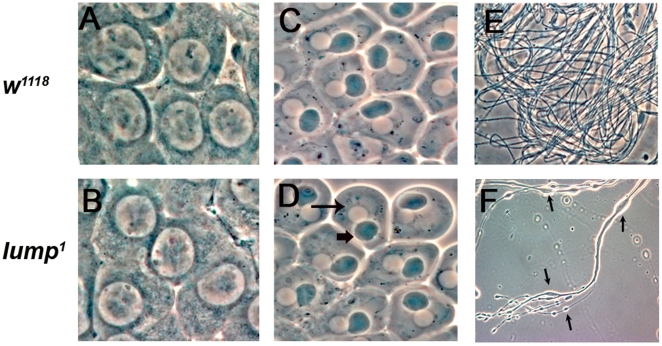
*lump* mutants are defective for spermatid individualization. (**A, B**) Testes squashes from 3-day old *w^1118^* and *lump^1^* mutant males showing primary spermatocytes with normal morphology. (**C, D)** Testes squashes from 3-day old onion stage spermatocytes from *w^1118^* and *lump^1^* mutant males have a single nucleus (arrow) and nebenkern (arrowhead) indicating no defects in meiosis. **E**. Testes squash from 3-day old *w^1118^* male showing mature motile sperm. **F**. Testes squashes from 3-day old *lump^1^* male showing clustered spermatids with elongated ‘lumps’. Spermatids are unable to individualize and have prominent blebbing (arrows).

To determine more precisely at what stage in spermatogenesis *lump^1^* mutants are defective we examined *lump^1^* mutant testes using phase contrast microscopy (Figure 4). Each early spermatid from *lump^1^* mutants has a single nucleus and associated mitochondrial nebenkern (Figure 4 A-D), indistinguishable from wild type controls. This indicates that the early stages of spermatogenesis, including mitosis and meiosis are proceeding normally in *lump^1^* mutants. However, we observed striking defects in spermatid individualization in the *lump^1^* mutants. The mutant testes contains syncytial spermatid bundles with irregular bulges and ‘lumps’ along the length of the sperm tail clusters (Figure 4F). This phenotype is typical for mutants defective for spermatid individualization [7]. We named the mutant ‘*lump’* to reflect the abundant persistent cytoplasmic bulges in the developing spermatids associated with this mutation.

**Figure 5:**
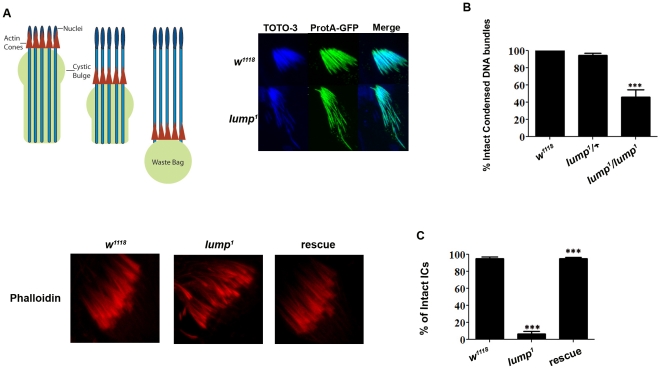
Spermatid individualization Defects in *lump^1^*. **A**. A cartoon of the actin-based individualization complex (illustrated by red triangles) drives the expulsion of unwanted cellular material from a syncytium of 64 closely associated spermatids. The cellular waste is collected in the cystic bulge (CB). At the end of the tail, the CB pinches off to release individual spermatozoa and form the waste bag (WB), which is subsequently degraded. *w^1118^* TOTO-3 DNA staining and protamine-GFP staining shows regular, aligned spermatid nuclei. *lump^1^* mutant TOTO-3 staining shows condensed spermatid nuclei, but with individual spermatids out of alignment with the others in the bundle. B. Quantification of the number of intact DNA bundles in *lump* mutant males compared to *w^1118^* and *lump^1^* heterozygotes. All data are presented as ± SEM. For each data set statistical significance was tested using ANOVA for independent observations. *** represents significant differences at P<0.0005. Testes stained with Alexa-Fluor-phalloidin (red) showing actin cones of the individualization complexes (ICs). Similar alignment defects are observed in *lump^1^* The IC in *lump^1^* mutants carrying a wild-type transgenic copy is restored. C. The number of intact actin cones in *lump^1^* mutant males is significantly reduced compared to *w^1118^* and *lump^1^* mutants carrying a rescuing transgene. All data are presented as ± SEM. For each data set statistical significance was tested using ANOVA for independent observations. *** represents significant differences at P<0.0001.

During the elongation phase in spermatogenesis, the spermatid nuclear bundles undergo morphological changes and the chromatin undergoes condensation. Immediately after meiosis, the spermatid nuclei are round, but as elongation progresses, the nuclei become canoe shaped, and finally, needle shaped. During these morphological changes, the 64 nuclei in the same bundle are aligned (Figure 5). In *lump^1^* mutant testes, the spermatid nuclei alignment is disrupted, and the nuclei are scattered and out of register (Figure 5B).

**Figure 6:**
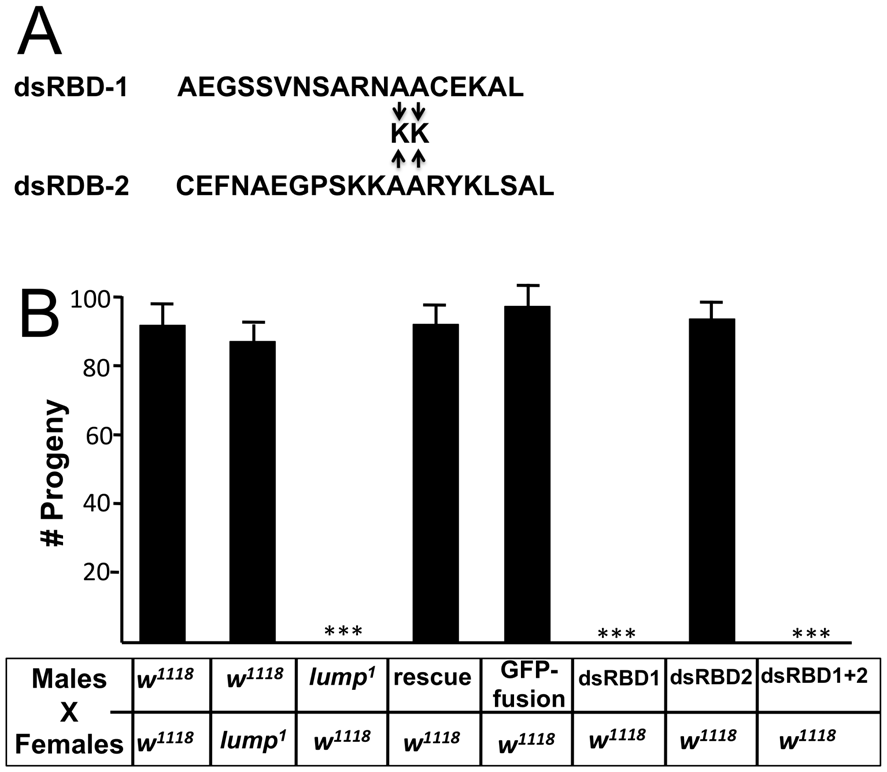
LUMP dsRBD-1 is required for LUMP male fertility function. **A**. The consensus dsRBD motifs for LUMP double-stranded RNA binding domain (dsRBD) are shown. Conserved hydrophobic alanine or leucine residues were replaced with basic lysines residues (K) in the first dsRBD, the second dsRBD, or both. **B**. The ability of the mutated LUMP constructs to rescue *lump^1^* male fertility was analyzed with fertility assays. *lump^1^* mutants carrying rescuing constructs with lesions in dsRBD1 or dsRBD1+2 are sterile. *lump^1^* mutants carrying rescuing constructs with lesions in dsRBD2 alone or GFP-LUMP are fertile. All data are presented as ± SEM. For each data set statistical significance was tested using ANOVA for independent observations. *** significant differences at P<0.0004.

In addition to the nuclear morphological changes that normally occur in late spermatid formation, the chromatin is also reorganized. There is a switch from histone-rich nucleosomes to a protamine-based structure [7]. Protamines are small, arginine rich, nuclear proteins that replace histones during the late canoe stage of nuclear elongation and condensation [25]. Protamines substitution is believed to be essential for sperm head condensation and DNA stabilization [26]. To investigate whether protamines abnormalities could explain the infertility in *lump^1^* we crossed a ProtamineA-GFP transgene into the *lump^1^* mutant background [27]. We find that ProtamineA-GFP is expressed and localized normally in *lump^1^* (Figure 6D). Therefore, *lump^1^* spermatid nuclei are misaligned but the DNA condensation process appears to be unaffected.

During spermatid individualization, intercellular bridges that join the 64 elongated syncytial spermatids are resolved by an actin-based structure called the individualization complex (IC) to produce mature spermatozoa (Figure 5A). This structure is composed of actin cones that assemble around the spermatid nuclei and migrate synchronously from the head to the tail of the spermatid bundles. This complex is thought to remove unnecessary organelles and excess cytoplasm from the spermatids, and resolve the syncytial spermatid bundles to individual spermatozoa. In wild type testes, actin cones in the individualization complex move in a synchronous manner from the heads of the 64 spermatids to the tips of their tails (Figure 5D). The initial formation of the IC appears normal in the *lump^1^* mutants. However, the actin complexes fail to translocate in a coordinated fashion. The individualization complexes become misaligned with stalled bundle migrations (Figure 5D). A wild-type transgenic copy of *lump* rescues the IC defect in *lump^1^* mutants (Figure 5D, E, Figure 6). These findings reveal that LUMP is required for proper translocation of the IC down the spermatids.

### LUMP dsRNA binding domain-1 is required for fertility

LUMP encodes a protein predicted to have 2 double-stranded RNA binding domains [18], [19]. To assess if either or both of these domains are required for LUMP function in the testes, we produced mutant versions of LUMP with lesions in these domains and assessed their function in spermatogenesis in vivo. We previously showed that replacing two hydrophobic residues with charged residues in the dsRNA binding domains of R2D2 abolished dsRNA binding and function [14]. We made the equivalent mutants in the dsRNA binding domains in genomic LUMP constructs, and introduced these transgenes into the *lump^1^* mutant background (Figure 6, Figure S2). The dsRNA constructs expressed LUMP proteins at levels comparable to wild type (not shown). Mutations in the second dsRNA-binding domain of LUMP did not alter the ability of the transgene to rescue fertility in the *lump^1^* mutant background (Figure 6). Therefore, this domain does not appear essential for LUMP function in the testes. However, mutations in the first dsRNA-binding domain alone, or in combination with lesions in the second, failed to rescue fertility. The spermatid bundles from these flies show the typical irregular actin cones and cytoplasmic blebs of *lump^1^* mutants (Figure 6B). These results indicate that LUMP dsRBD-1 is essential for LUMP function in male fertility.

## Discussion

We show here that LUMP, a protein predicted to encode two double-stranded RNA binding domains (18, 19), is a previously unknown regulator of the late stages of male germ cell development. Mutations in *lump* are the direct cause of the male fertility defects, as the defects are reverted by precise excision or germline rescue. We also show this protein has a developmentally regulated nuclear to cytoplasmic localization shift in male germ cells. We find LUMP expression is not restricted to the testes, but this tissue appears to be the most sensitive to reduced LUMP function.

*lump^1^* mutants do not show obvious defects in RNAi, miRNA or stellate suppression, but since *lump^1^* is not a null, we cannot rule out the possibility that residual LUMP expression is sufficient for these functions. We note that homozygous *lump^1^* mutants are rare compared to their balanced sibs (not shown), suggesting LUMP also affects viability. This is consistent with the general expression pattern we observe for this gene. The most striking defect in *lump^1^* mutants, however, is sterility arising from abnormal late stage spermatid development. This phenotype is distinct from previously described germ cell processes regulated by RNAi-like mechanisms that impact the early steps in this process [28], [29].

Mutants in multiple RNAi components are known to have defects in sperm development, but all are distinct from those observed in *lump^1^*. These genes include *piwi*, *ago-3*, *aubergine*, *loquacious*, and *spindle-E*. *piwi* and *ago-3* are required for germline stem cell maintenance and transposon silencing in the testes. The testes in *piwi* mutants are small because germ line stem cells fail to renew after division resulting in loss of germline stem cells [8]. *lump^1^* testes do not have defects in germline stem cell maintenance. *loquacious, aubergine*, *spindle-E,* and *ago-3* are important for silencing the *Stellate* locus. Mutations in these genes result in de-repression of *Stellate* and Stellate crystal formation in the testes resulting in infertility [30]. We observed no crystals in *lump^1^* mutant testes. Thus LUMP has a role in male spermatogenesis that is distinct from these previously characterized RNAi-like male fertility mechanisms.

In *lump^1^* mutants, spermatid individualization, essential for production of mature motile sperm, is defective. Normally, individualization is initiated when actin cones assemble around the spermatid nuclei [3], [4] [31]. The actin cones move synchronously along all 64 spermatids in a cyst toward the spermatid tails. This produces a visible cystic bulge that ultimately forms the waste bag when it reaches the termini of the spermatid tails [3], [4] (Figure 6A). This process eliminates the cytoplasmic bridges and removes excess cytoplasm and unneeded organelles from the spermatids. *lump^1^* mutants are defective for the migration of the individualization complex, producing spermatids that remain tethered together, resulting in infertility.

The individualization defects are not likely to result from the direct action of LUMP. Introduction of transgenic GFP-tagged LUMP in a *lump^1^*mutant background fully rescues *lump^1^* mutant function. Strikingly, GFP-LUMP does not localize to the individualization complex. Therefore, it is likely LUMP acts to regulate expression or localization of other factors directly involved in individualization, and loss of this function produces sterility. For example, if LUMP acts in a novel RNAi-like pathway, it may function to suppress a subset of transcripts which when inappropriately expressed, produce individualization defects. Indeed, a number of genes produce individualization defects when mutated, including Dronc, Drice, and Hid proteins that are expressed in elongated spermatids and are utilized to promote spermatid individualization [32]. Furthermore, mutations in cytoskeleton proteins, *ddlc1* (*dynein light chain 1), myosin VI (jar1), and myosin V (MyoV^Q1052st^)* cause scattered spermatid nuclear bundles and a disruption in actin cone movement leading to sterility in these mutants [33], [34] [35]–[36]. Similarly GLD2, a poly-A polymerase, has individualization defects similar to those of *lump^1^*, but also has protamine condensation defects [37]. Importantly, these phenotypes are observed in loss-of-function alleles of these genes. By contrast, if LUMP is a negative regulator of gene expression, we expect sterility to result from excess or inappropriate expression of genes. However, it is possible LUMP acts indirectly by repressing an inhibitor of one or more of these known individualization factors. Alternatively, LUMP may not function in an RNAi-like mechanism, but instead might be required for proper localization or transcriptional regulation of one or more transcripts that have double-stranded or RNA hairpin features. For example, Staufen is a dsRBP required for mRNA localization and translational repression in the germline [38]. LUMP may function in a similar manner.

Our work supports the findings of Gerbasi et al. who independently studied the *lump^1^* mutant, which they named *blanks* [16]. These workers identified LUMP as a siRNA-binding protein in S2 extracts associated with a unique protein complex. In their hands, RT-PCR experiments and anti-LUMP antiserum also identified this protein as testes enriched. We find more general expression of *lump* in various adult tissues including the adult brain (Figure 6 and Figure S1) but concur that LUMP is most strongly expressed in the testes. Our work extends Gerbasi's findings by revealing the first but not the second predicited dsRNA-binding domain is critical for LUMP function in vivo and by demonstrating the defects observed in *lump^1^* result directly from the transposable element, and not from other genes potentially defective in this strain. In addition, we were able to observe GFP-LUMP in living flies, detecting dynamic subcellular localization of this protein and lack of LUMP in the individualization complex. We observe GFP-LUMP in the nucleus of spermatocytes, but translocation of LUMP-GFP to the cytoplasm at the early spermatid stage. This localization may be important for transporting specific transcripts to the cytoplasm or inhibiting their transport or translation at earlier stages. Gerbasi et al. identified several genes with increased expression in *lump^1^* mutants. However, it is not clear any of these candidates are responsible for the individualization defects. Identifying the RNA molecules that bind to LUMP in testes will provide important clues about possible gene targets regulated by LUMP.

## Materials and Methods

### Fly Stocks

The CG10630^KG00804^ (*lump^1^* mutant) fly stock was obtained from the Bloomington Stock Center. *UAS-ProtamineA-GFP* flies [27] were kindly provided by John Belote (Syracuse University).

### Transgenic Animals

A 6 kb genomic BamHI-Asp718 fragment containing 4kb upstream of the *lump* initiation methionine, the coding sequence and 1 kb of sequence downstream of the putative polyadenylation site of the *lump* reading frame was cloned into pCasper4 [39]. Germline transformants were generated by standard procedures [40]. A transgenic strain with the rescuing P element integrated into the second chromosome was crossed into the *lump* mutant background for rescue experiments.

LUMP dsRNA binding mutants were produced by introducing point mutations in the rescuing construct with PCR primers. All PCR fragments were sequenced to confirm only the desired changes were introduced. LUMP-GFP fusion transgenes were produced by introducing a Not1 site after the lump ATG and inserting a GFP cDNA in frame to produce a GFP-LUMP fusion.

Revertants were produced by crossing *lump^1^* to a transposase stock [41] using standard genetic techniques.

### Fertility Assay

Three 3 day-old virgin males and females were placed in culture vials for 5 days at 25°C, at which time the parental flies were removed. The resulting F1 progeny were counted. Triplicate vials were set up for each genotype, and the assay was repeated at least three times. For each data set the statistical significance was tested using ANOVA for independent observations.

### Testes Cytology Analysis

Testes were dissected from 2–3 day adult males in *Drosophila* Ringers solution with Pipes, squashed between a coverslip and slide and examined directly by phase-contrast microscopy as previously described [42].

### Western immunoblots and immunocytochemistry

Protein samples from wild type and mutant tissues were analyzed on 12% SDS-polyacrylamide gels [43] and transferred to nitrocellulose membrane (Optitran BA-S 83, S&S, Dassel, Germany) using a semi-dry blotter (Bio-Rad). LUMP was detected with anti-LUMP antiserum (see below).

For DNA and F-actin staining, testes from 2–3 day old adults were dissected in phosphate-buffered saline (PBS, 130 mM NaCl, 7 mM Na_2_HPO_4_, 3 mM NaH_2_PO_4_, pH 7.2) and then fixed with EM grade 4% paraformaldehyde (Electron Microscope Sciences) in PBST (PBS+0.3% Triton X-100) for 30 minutes at room temperature. After washing with PBST three times, the testes were blocked with 4% normal goat serum (NGS) in PBST at 4^o^ C overnight, washed once in PBST, twice in PBS, and incubated with 4 units/mL of Alexa Fluor 546 phalloidin (Molecular Probes) for 4 hours at room temperature. The testes were rinsed three times in PBS, and mounted in VectaShield mounting medium (Vector Labs). Fluorescent images were obtained using a Zeiss LSM 5 confocal microscope.

### Generation of LUMP antibody

The LUMP antiserum was raised against a peptide corresponding to the amino-terminal-15 amino acids as described previously [44].

## Supporting Information

Figure S1 **Expression of** ***lump*** **in wild type tissues. A. RT-PCR detects lump expression in most tissues.** A 240 base-pair fragment was amplified from cDNAs produced from various tissues and developmental stages. M, HaeIII digested PhiX174 markers. 0–2, 0–2 hour old embryos. 2, second instar larvae. 3, third instar larvae. WP, white pupae. DP, dark pupae. A, antenna. H, head. B, bodies. F, Whole female. T, Testes. C, male carcuses after removal of the testes. **B. Frozen tissue section through the head of a** ***lump^1^*** **mutant carrying GFP-LUMP expressed by its own promoter.** Many tissues, including the lighter cells in the CNS above, express GFP in the cell bodies.
(TIFF)

Figure S2 **Alignment of Lump homologs from three Drosophila species.** Clustal alignment of *Drosophila melanogaster* LUMP protein sequence with homolgous proteins from *Drosophila sechelia* and *Drosophila virilus*. Boxes denote predicted dsRNA binding domains (PFAM, Sanger Institute, UK). _*_ denotes conserved small hydrophobic residues important for binding RNA.
(TIF)

Methods S1 **Supporting methods.**
(DOCX)
