# Supplementary figures and images for: LUMP Is a Putative Double-Stranded RNA Binding Protein Required for Male Fertility in Drosophila melanogaster
Source: PLoS One. 2011 Aug 30;6(8):e24151. doi: 10.1371/journal.pone.0024151 (PMC3166160; doi:10.1371/journal.pone.0024151)

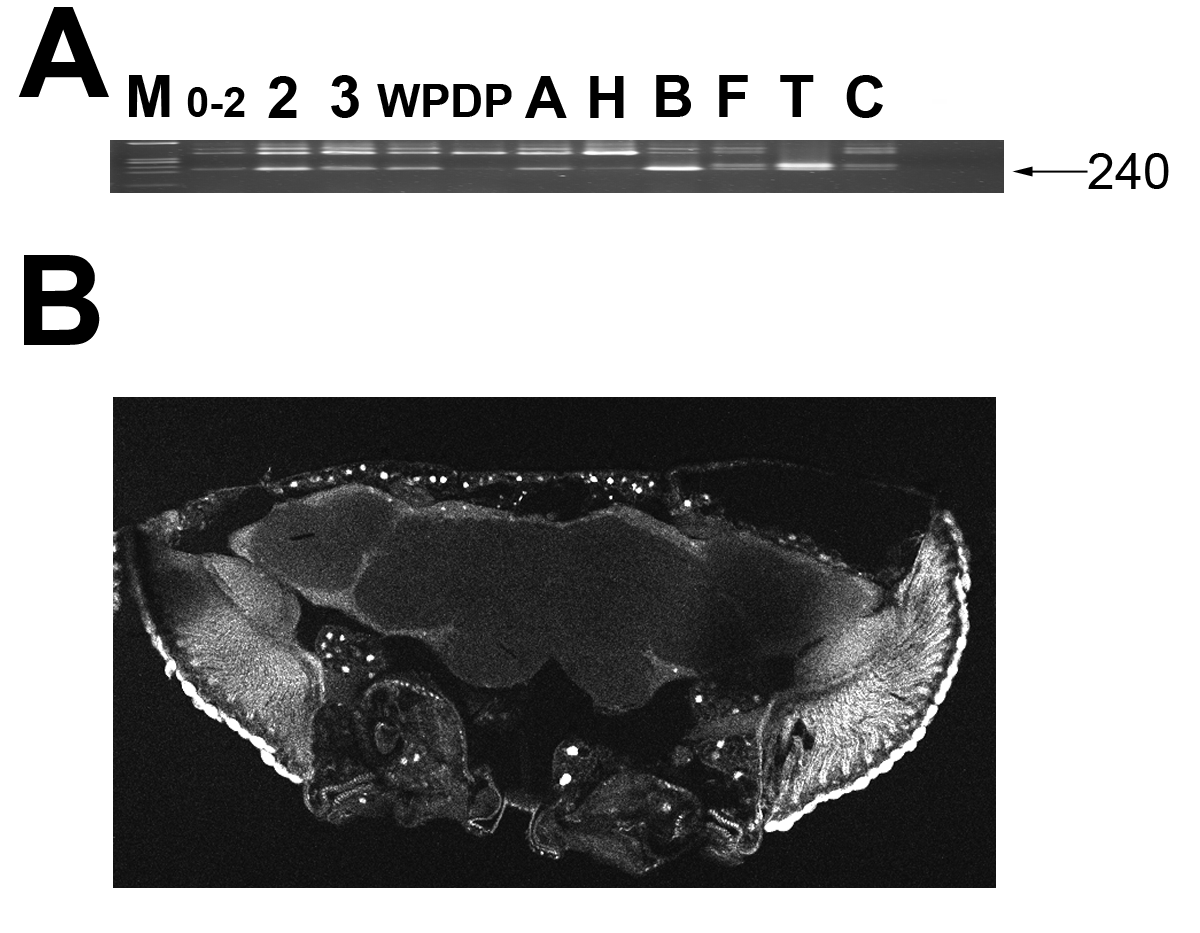

Supplement: Figure S1 — Expression of lump in wild type tissues. A. RT-PCR detects lump expression in most tissues. A 240 base-pair fragment was amplified from cDNAs produced from various tissues and developmental stages. M, HaeIII digested PhiX174 markers. 0–2, 0–2 hour old embryos. 2, second instar larvae. 3, third instar larvae. WP, white pupae. DP, dark pupae. A, antenna. H, head. B, bodies. F, Whole female. T, Testes. C, male carcuses after removal of the testes. B. Frozen tissue section through the head of a lump1 mutant carrying GFP-LUMP expressed by its own promoter. Many tissues, including the lighter cells in the CNS above, express GFP in the cell bodies. (TIFF) [file pone.0024151.s001.tiff]

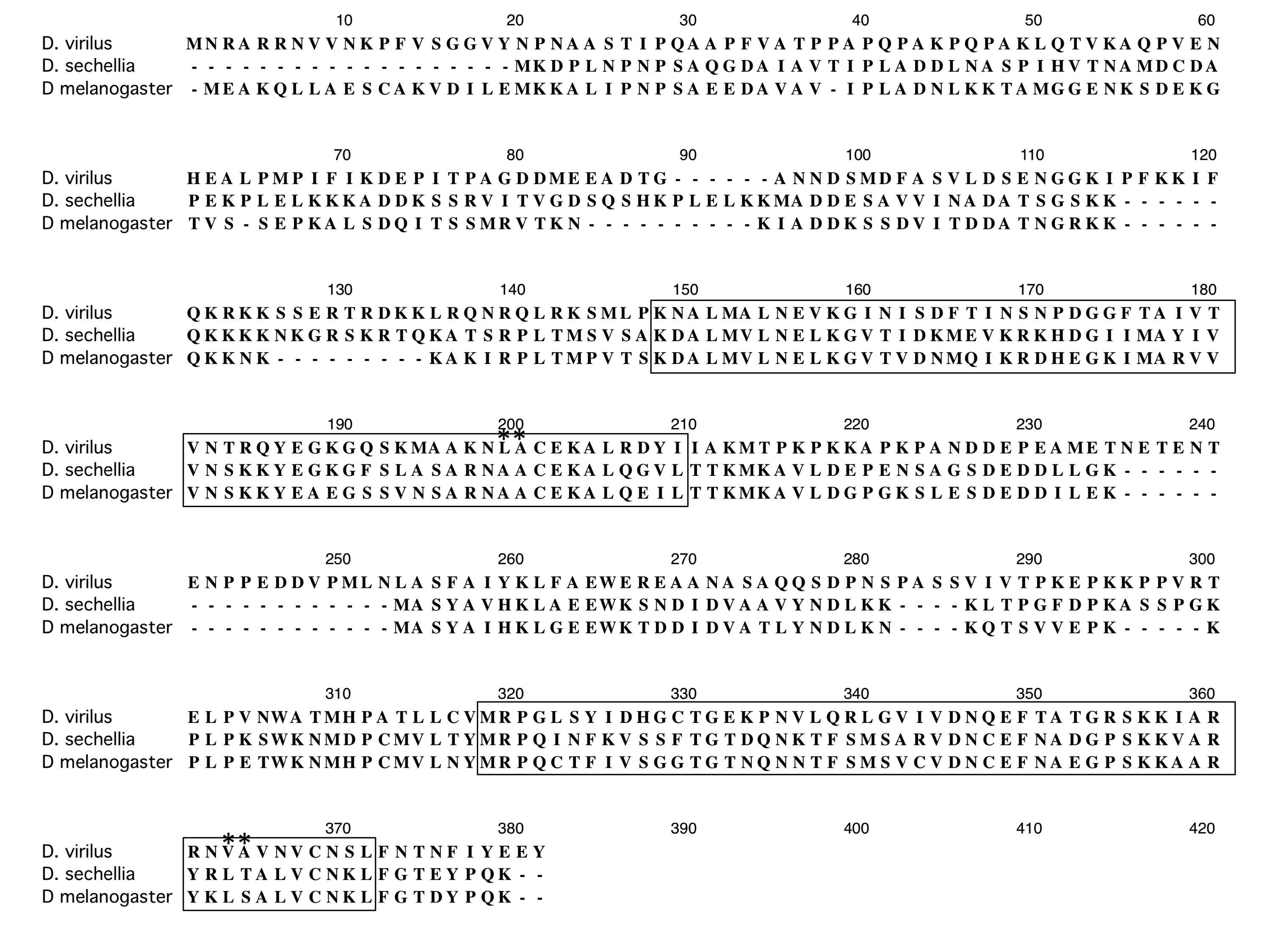

Supplement: Figure S2 — Alignment of Lump homologs from three Drosophila species. Clustal alignment of Drosophila melanogaster LUMP protein sequence with homolgous proteins from Drosophila sechelia and Drosophila virilus. Boxes denote predicted dsRNA binding domains (PFAM, Sanger Institute, UK). * denotes conserved small hydrophobic residues important for binding RNA. (TIF) [file pone.0024151.s002.tif]
