# Supplementary material for: LUMP Is a Putative Double-Stranded RNA Binding Protein Required for Male Fertility in Drosophila melanogaster
Source: PLoS One. 2011 Aug 30;6(8):e24151. doi: 10.1371/journal.pone.0024151 (PMC3166160; doi:10.1371/journal.pone.0024151)
Supplement: Methods S1 — Supporting methods. (DOCX) [file pone.0024151.s003.docx]

**Methods S1**

RT-PCR

1 ug of total RNA isolated from the indicated tissues was used to generate cDNA using Superscript III as recommended by the supplier (InVitrogen, Carlsbad, CA). Primers spanned the 169 BP intron in *lump* to distinguish genomic DNA products. Primers used were Forward 5’-GCAAAGGTGGACATCTTAGAG-3’ and Reverse 5’-CGTCAGAAGATTTATCATCGGC. Products were resolved on a 3% Nusieve GTG agarose gel (Cambrex, Berkshire, UK). 10 um frozen tissue sections (Bright cryostat, Cambridgeshire, UK) were cover-slipped in glycerol and observed directly under confocal microscopy (Zeiss LSM 510).
